# The effect of in-hospital follow-up on early post-transfer distress in families of patients in pediatric intensive care unit

**DOI:** 10.3389/fmed.2025.1706926

**Published:** 2026-01-14

**Authors:** Dan Peng, Jianxiong Peng, Xia Wu, Yuanna Liu, Zhaohong He, Min Hu, Tingting Huang, Yanhong Chen, Yufan Yang, Meihua Liu, Xinping Zhang

**Affiliations:** 1 Department of Intensive Care Unit, The Affiliated Children’s Hospital of Xiangya School of Medicine, Central South University (Hunan Children’s Hospital), Changsha, Hunan, China; 2 School of Nursing, Hunan University of Chinese Medicine, Hanpu Science and Education Industrial Park, Changsha, Hunan, China; 3 Medical Insurance Center, The Affiliated Children’s Hospital of Xiangya School of Medicine, Central South University (Hunan Children’s Hospital), Changsha, Hunan, China

**Keywords:** C-PICSQ, early depression, family members, inpatient follow-up, PICU (Pediatric Intensive Care Unit)

## Abstract

**Objective:**

To explore the effect of in-hospital follow-up on post-intensive care syndrome in families of PICU (Pediatric Intensive Care Unit) patients.

**Methods:**

A total of 88 families of children admitted to the PICU of Hunan Children’s Hospital from January 1, 2023, to September 30, 2023, were selected as research subjects. The control group received routine nursing intervention, while the experimental group, based on the control group’s care, established a PICU communication team. The team was divided into three subgroups: the department director and head nurse, Chief resident and Responsible Nurse Team Leader, Doctor in charge of the patient’s bed and Person in charge of health education. On the day of Transfer to another department and 3 days after Transfer to another department, one group of personnel communicated face-to-face with the family members regarding the patient’s condition and precautions. After the communication, the intervention effects on the parents of the children in both groups were assessed within 30 min using the Post-Intensive Care Syndrome Questionnaire (C-PICSQ), the Depression-Anxiety-Stress Scale (DASS-21), and the Critical Care Family Satisfaction Scale (CCFSS).

**Measurements and main results:**

Before the intervention, there were no statistically significant differences in the C-PICSQ scores, DASS-21 scores, and CCFSS scores between the family members of children in the two groups (*p* > 0.05). Three days after Transfer to another department, the C-PICSQ score of 16.25 ± 3.93 for the family members in the experimental group was lower than the C-PICSQ score of 33.25 ± 5.97 in the control group. The DASS-21 score of 17.91 ± 2.18 for the experimental group was lower than the DASS-21 score of 34.77 ± 5.30 for the control group, and the CCFSS score of 80.91 ± 9.64 for the experimental group was higher than the CCFSS score of 37.89 ± 14.49 for the control group, with all differences being statistically significant (*p* < 0.05).

**Conclusion:**

In-hospital follow-up can effectively alleviate post-intensive care unit (PICU) syndrome in family members, reduce negative emotions such as depression, anxiety, and stress among parents, improve family satisfaction.

## 1 Introduction

In China, most Pediatric Intensive Care Units (PICU) operate under a closed, non-visiting management model. Due to the unique nature of the environment, children need to face unfamiliar treatment settings and healthcare personnel alone, which often leads to feelings of fear, crying, silence, and non-cooperation. Additionally, the characteristics of critically ill children, such as rapidly changing conditions and high mortality rates, further complicate the situation (1). The family members of the child may experience negative emotions such as tension, anxiety, fear, pessimism, and despair due to concerns about the child not receiving proper care, uncertainty about the child’s prognosis, the unfamiliar medical environment, separation from the child, unclear treatment outcomes, inability to be with the child at all times, self-blame, guilt, and medical expenses. Related studies have reported that the incidence of negative emotions among the family members of children in the PICU can be as high as 98.4% (2). In severe cases, these emotions can even affect the doctor-patient relationship, which in turn may impact the effective treatment of the child (3). Post-intensive care syndrome family (PICS-F) refers to a constellation of persistent psychological, physiological, and cognitive impairments experienced by family members during the child’s ICU stay or after transfer. Early depression in family members manifests as a state of depressed mood, frequent crying, fatigue, and helplessness in the initial period (typically ranging from days to weeks) after the child is moved to a general ward or rehabilitation unit, which can cause by a sudden shift of responsibility, a “delayed fear” reaction, environmental changes, uncertainty, and physical/mental exhaustion. Early depression in family members represents an acute, situational emotional response that may serve as a precursor and warning sign for the subsequent development of PICS-F. Early identification and psychological support during this critical window are crucial, as they may potentially mitigate or prevent the onset of full-blown PICS-F. How to alleviating negative emotion for family members and improving nursing satisfaction are urgent issues that need to be addressed. To implement the “Healthy China 2030” Planning Outline, which emphasizes that hospitals should strengthen humanistic care in medical services and build a harmonious doctor-patient relationship (4). Currently, both domestic and international research has primarily focused on Post-Intensive Care Syndrome in children (Post-intensive care syndrome pediatric, PICS-p). However, studies on Post-Intensive Care Syndrome in family members (Post-intensive care syndrome family, PICS-f) and in-hospital follow-up are rare. Therefore, our department has implemented in-hospital follow-up for early depression in family members after a child’s transfer from the PICU. This initiative involves an expert team led by the department head and head nurse, who provide early intervention for early depression in family members. The intervention was delivered on the day of the patient’s transfer from the PICU and 3 days post-transfer. This approach has further strengthened the trust between medical staff, the children, and their families, maximizing cooperation from both patients and families. It has alleviated PICS-f, reduced negative emotions such as early depression, anxiety, and stress in family members, and improved the overall satisfaction of families with the care provided. The report is as follows.

## 2 Study subjects and methods

### 2.1 Subject investigated

The study subjects were family members of children admitted to the PICU (Pediatric Intensive Care Unit) at Hunan Children’s Hospital from January 1, 2023, to September 30, 2023. Inclusion criteria: ① The family members of the children provided informed consent and were willing to participate in the study; ② The family members of children admitted to the PICU; ③ The family members were required to care for the child for ≥40 h/week and more than 4 h per day. Exclusion criteria: ① The child was admitted to the PICU for <24 h; ② The child withdrew from treatment or mortality; ③ The family members had intellectual disabilities or mental illnesses that prevented them from cooperating with the survey.

According to the literature (5), the sample size was calculated using PASS 15.0 software, Family satisfaction in the intensive care u1=0.73,u2=0.82,σ=11 , two-tailed test *α* = 0.05 and 1−*β* = 0.95. The required total sample size for this study was calculated to be 80 cases. Considering a 10% Lost visit rate, the final required sample size was confirmed to be 88 cases. In this study, a total of 88 samples were included, with 44 cases in each group, meeting the relevant requirements. The general data of the two groups of children and their families were statistically analyzed, and no significant differences were found (*p* > 0.05), indicating comparability, as shown in Table 1. This study has been approved by the hospital’s ethics committee, with the ethics approval number HCHLL-2025-04.

**Table 1:** Comparison of general information between children and their families.

Project	Classification	Experimental group	Control group	t/X^2^	*P*
		(*n* = 44)	(*n* = 44)		
Child’s gender (%)	Man	23 (52.27)	17 (38.64)	1.282	0.154
	Woman	21 (47.73)	27 (61.36)		
Child’s age (%)	<6 years old	25 (56.82)	22 (50.00)	0.770	0.512
	6–12 years old	15 (34.09)	16 (36.36)		
	13 years old or older	4 (9.09)	6 (13.64)		
Only child status (%)	Yes	16 (36.36)	18 (40.91)	0.433	0.396
	No	28 (63.64)	26 (59.09)		
Parents’ ages (%)	20–30	8 (18.19)	14 (31.82)	1.112	0.665
	31–40	20 (45.45)	16 (36.36)		
	41–50	16 (36.36)	14 (31.82)		
Relationship with the child (%)	Father	9 (20.45)	10 (22.73)	0.366	0.503
	Mother	31 (70.45)	27 (61.36)		
	Grandfather/Grandmother	4 (9.10)	7 (15.91)		
Marital status (%)	Unmarried, divorced, widowed	4 (9.10)	7 (15.91)	0.961	0.054
	Married	40 (90.90)	37 (84.09)		
Level of education (%)	High school or below	25 (56.82)	23 (55.27)	0.424	0.449
	High school or above	19 (43.18)	21 (47.73)		
Occupational status (%)	Unemployed	16 (36.36)	17 (38.64)	0.218	0.665
	Employed	28 (63.64)	27 (61.36)		
Number of children (%)	One	14 (31.82)	16 (36.36)	0.000	0.388
	Two	23 (55.27)	19 (43.18)		
	Three or more	7 (15.91)	9 (20.45)		
Residence (%)	Rural	16 (36.36)	18 (40.91)	0.433	0.396
	Town	28 (63.64)	26 (59.09)		
Payment for hospitalization expenses (%)	Self-funded	9 (20.45)	10 (22.73)	0.235	0.660
	Health insurance	34 (77.27)	33 (75.00)		
	Third-party responsible person	1 (2.27)	1 (2.27)		
Monthly household income (%)	Less than 3,000 yuan	12 (27.27)	11 (25.00)	0.443	0.905
	3,000–6,000 yuan	22 (50.00)	21 (47.73)		
	More than 6,000 yuan	10 (22.73)	12 (27.27)		
First hospitalization (%)	Yes	29 (65.91)	28 (63.64)	0.221	0.661
	No	15 (34.09)	16 (36.36)		

### 2.2 Research methods

#### 2.2.1 Control group adopt conventional interventions

The content mainly includes: ① On the day of admission, Medical staff in charge of bed staff will introduce themselves, inquire about the child’s condition, inform about the visiting system and precautions, etc. ② The department has designated Mondays, Wednesdays, and Fridays as “Doctor-Nurse Listening Days.” Special family members can adjust the listening time according to their needs, with doctor in charge of the patient’s bed and Nurse. Team Leader to listen to the family’s opinions, This is to further understand the difficulties faced by the families of patients, and based on their needs, in conjunction with the existing rules and regulations of the hospital department, to alleviate the family’s concerns, improve work and service attitudes, solve practical problems, and build trust. ③ Short videos were sent for 1 ~ 3 min every day, and older children were visited for 5 ~ 10 min. ④ On the day of Transfer to general department, doctor in charge of the patient’s bed and the duty nurse will communicate the precautions for the patient’s transfer.

#### 2.2.2 Experimental group

A communication team of 8 members was established based on the control group for the PICU, consisting of the department director, head nurse, chief resident, responsible nurse team leader, doctor in charge of the patient’s bed, Person in charge of health education each 1, and 2 nursing graduate students. The department head serves as the team leader, the head nurse as the deputy leader, and the remaining members are team members. The team leader is responsible for communication regarding medical treatment, the deputy leader handles communication related to nursing, team members communicate with the family regarding the child’s treatment and nursing needs in the absence of the department head or head nurse, and the nursing graduate is responsible for the collection and collation of data.

##### 2.2.2.1 In-hospital follow-up

**Figure 1:**
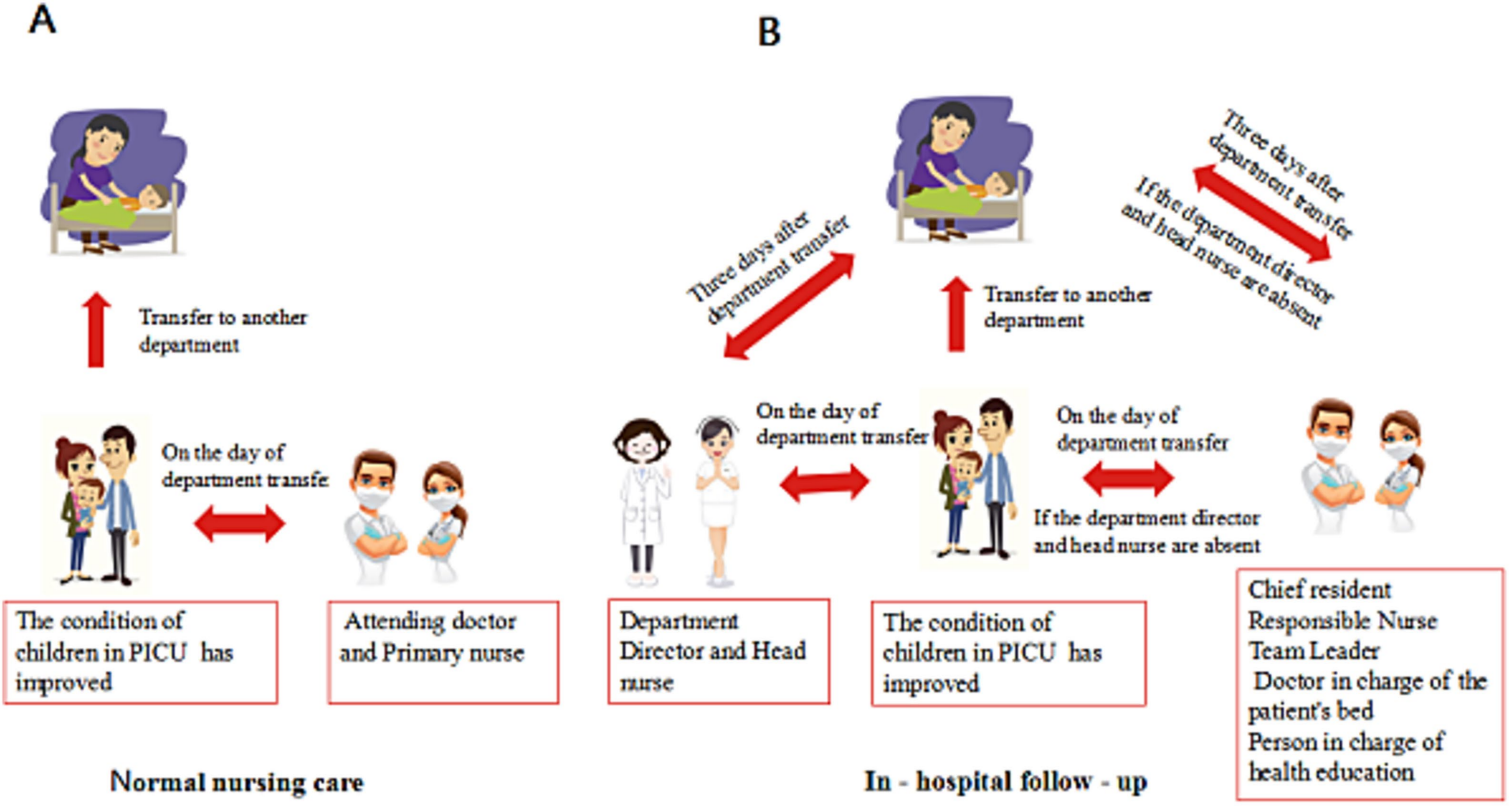
Comparison of routine nursing and in-hospital follow-up.

① Assessment and Plan: Children and their families may experience functional impairments in physiological, cognitive, and psychological aspects during and after transfer from the PICU, which can manifest as anxiety, depression, insomnia, and other symptoms of Post-Intensive Care Syndrome (PICS). Individualized follow-up after PICU discharge is beneficial for the early identification of early depression in family members. Before the child is transferred, an individualized follow-up plan should be developed based on the child’s specific condition, including the follow-up schedule, frequency, and content. ② Nursing Safety Practice: Follow-up should occur at the time of the child’s On the day of Transfer to another department from the PICU and again 3 days after Transfer to another department; The follow-up work is the responsibility of the department director and the head nurse. In their absence, the chief resident and responsible nurse team leader will carry out the follow-up. If both the chief resident and team leader are absent, the doctor in charge of the patient’s will take in charge of health education will handle the follow-up, as shown in Figure 1. ③ Education and Support: During the follow-up process, the follow-up personnel are responsible for educating the child, family, and ward doctors and nurses. This education is delivered through oral instructions, bedside demonstrations, communication, and other methods to teach critical care-related knowledge and skills, answer questions related to the children’s condition, and provide psychological support. This also helps to enhance the confidence and competence of the ward’s attending physicians and nurses in managing children transferred from the PICU. ④ Communication and Collaboration: PICU nursing follow-up personnel can collaborate with the ward’s responsible nurse to assess the child, identify nursing issues, develop a nursing plan, and have the ward’s responsible nurse supervise its implementation, thereby ensuring the continuity of care during the transition period for children transferred from the PICU.

##### 2.2.2.2 Training and assessment

Training for team members in the early stage includes: ① Definition, Influencing Factors, and Interventions of Family Post-ICU Syndrome. ② The Importance and Significance of In-Hospital Follow-Up for Children and Their Families, Standardizing Language and Techniques for Communication with Families, Actively Engaging in Communication with Children and Their Families, and Enhancing Parental Involvement. ③ Effect assessment tool: The assessment process and methods for the Family Post-ICU Syndrome Questionnaire (C-PICSQ), the Emotional Self-Assessment Scale/Depression-Anxiety-Stress Scale (DASS-21), and the Family Satisfaction with Critical Care Scale (CCFSS). Team members can begin in-hospital follow-up only after passing the assessment.

### 2.3 Evaluating indicator

#### 2.3.1 General survey

The study used a self-designed questionnaire to collect demographic and other factors, including: the child’s gender, age, whether they are an only child, the child’s diagnosis, length of hospitalization, and the caregiver’s gender, age, relationship with the child, number of children, marital status, education level, occupation, monthly income, and method of payment for hospitalization expenses.

#### 2.3.2 Post-Intensive Care Syndrome Questionnaire, C-PICSQ (Chinese version)

The scale was developed by Korean scholar Yeon et al. (6), and later revised through localization by domestic scholar Sun Tingting. The scale includes three dimensions: cognitive dimension (3 items), physiological function dimension (4 items), and psychological dimension (5 items), totaling 12 items. It is graded on a 4-point Likert scale: 0 (never) to 3 (always). The total score ranges from 0 to 54, with higher scores indicating more severe symptoms. The overall Cronbach’s *α* coefficient of the scale is 0.827.

#### 2.3.3 Mood Self-Assessment Scale/Depression-Anxiety-Stress Scale (DASS-21)

The scale (Chinese version) was developed and revised by Hong Kong scholar Taouk (7), and the Simplified Chinese version was adapted by Gong Xu et al. The scale consists of three subscales, each containing 7 items. These include the Depression subscale (items 3, 5, 10, 13, 16, 17, 21), the Anxiety subscale (items 2, 4, 7, 9, 15, 19, 20), and the Stress subscale (items 1, 6, 8, 11, 12, 14, 18), with a total of 21 items. It is graded on a 4-point Likert scale: 0 (not applicable) to 3 (most applicable). The total score ranges from 0 to 42, with higher scores indicating more severe levels of depression, anxiety, or stress. The overall Cronbach’s *α* coefficient of the scale is 0.84.

#### 2.3.4 The family satisfaction scale of critical risk patients (Critical Care Family Satisfaction Survey, CCFSS)

This scale was introduced by Wasser et al. (8), who standardized the version of the Family Satisfaction with Critical Care (C-CCFSS) scale, originally developed for families of critically ill patients abroad. The Chinese version has demonstrated high reliability and validity, and is more suitable for Chinese expressions. The C-CCFSS consists of 27 items, divided into five dimensions: Information Acquisition (7 items), Assurance of Condition (7 items), Acceptance (3 items), Support (6 items), and Comfort (4 items). Each item is scored from 1 to 5, ranging from “very dissatisfied” to “very satisfied,” with a total score of 15. The higher the score, the higher the level of satisfaction among ICU patients’ families. As one of the tools for evaluating ICU family satisfaction, this scale has proven to be reliable, with a Cronbach’s *α* coefficient of 0.929 for its internal consistency.

### 2.4 Data collection method

Data on the general information of the children, as well as their families’ scores on the Critical Care Post-Intensive Care Syndrome Questionnaire (C-PICSQ), the Depression-Anxiety-Stress Scale (DASS-21), and the Critical Care Family Satisfaction Survey (CCFSS), were collected and compiled by two trained nursing master’s students. Family interviews were conducted on the day of the child’s transfer to the department and again 3 days after the transfer. The communication team jointly evaluated, collected, and organized the data during these two time points.

### 2.5 Statistical method

The data were analyzed using SPSS 25.0 statistical software. Continuous variables were described as mean ± standard deviation and analyzed using analysis of variance (ANOVA). Categorical variables were described using frequency and composition ratio, and analyzed using the chi-square test. The comparison between two groups was performed using an independent samples *t*-test. The severity scores of clinical symptoms at different time points were analyzed using repeated measures analysis of variance. A *p*-value of less than 0.05 was considered statistically significant.

## 3 Results

### 3.1 Comparison of general information between the two groups of children and their families

**Figure 2:**
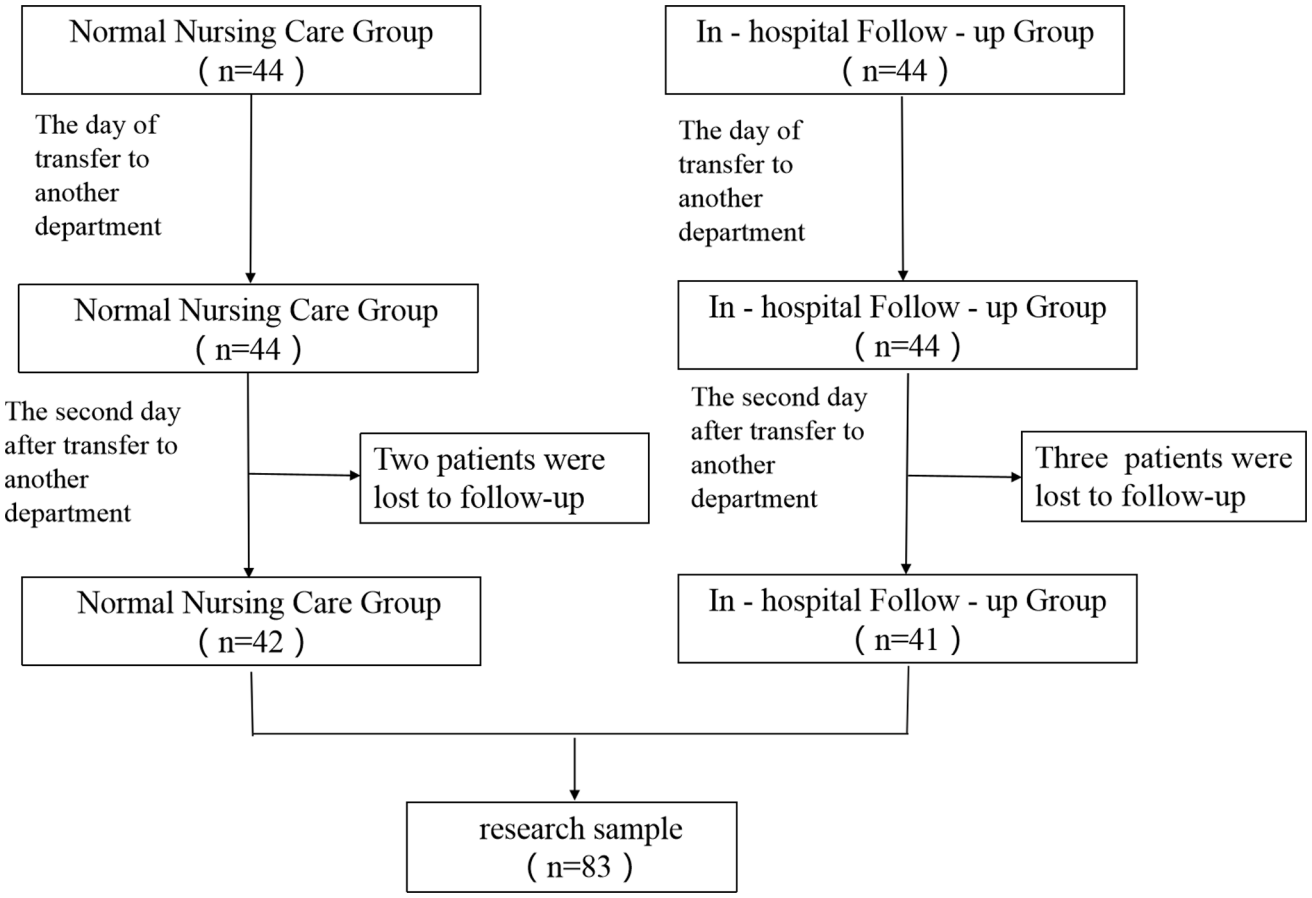
Loss to of family members in the two groups to follow-up.

A total of 88 children were included in this study, with 44 children in each group. There were no statistically significant differences between the two groups in terms of gender, age, duration of hospitalization, or whether the child was an only child (*p* > 0.05), as shown in Table 1. During the study, both groups of children experienced loss to follow-up: on the second day after the transfer, 2 children in the control group and 3 children in the experimental group requested to be discharged and were lost to follow-up, as shown in Figure 2.

### 3.2 Comparison of C-PICSQ scores between the families of the two groups of children

There was no statistically significant difference in the C-PICSQ scores of the families of the two groups of children before the intervention (*p* > 0.05). After the intervention was implemented through hospital-based follow-up, on the day of transfer, the C-PICSQ score of the families of the children in the experimental group (20.16 ± 4.05) was lower than that of the control group (31.66 ± 6.74), and the difference was statistically significant (*p* < 0.05). On the third day after the transfer, the C-PICSQ score of the families of the children in the experimental group (16.25 ± 3.93) was lower than that of the control group (33.25 ± 5.97), and the difference was statistically significant (*p* < 0.05), as shown in Table 2.

**Table 2:** Comparison of C-PICSQ scores before and after the intervention among the families in the two groups $(\bar{X}+s,$ points).

Group	Before the intervention	On the day of transfer	On the third day following the transfer	Intergroup effects	Time effect	Interaction effect
				*F*	*F*	*F*
Experimental group	24.48 ± 6.99	20.16 ± 4.05	16.25 ± 3.93	91.665	8.107	41.122
Control group	26.57 ± 7.38	31.66 ± 6.74	33.25 ± 5.97			
*t*	−1.364	−10.138	−23.866			
*P*	0.257	0.007	<0.001			

### 3.3 Comparison of DASS-21 scores before and after intervention in the families of the two groups of children

Before the intervention, there was no statistically significant difference in the DASS-21 scores between the families of the two groups of children (*p* > 0.05). After the intervention was implemented through hospital-based follow-up, on the day of transfer, the DASS-21 score of the families of the children in the experimental group (23.23 ± 3.26) was lower than that of the control group (32.20 ± 4.78), and the difference was statistically significant (*p* < 0.05). On the third day after the transfer, the DASS-21 score of the families of the children in the experimental group (17.91 ± 2.18) was lower than that of the control group (34.77 ± 5.30), and the difference was statistically significant (*p* < 0.05) (see Table 3).

**Table 3:** Comparison of DASS-21 scores before and after the intervention among the families in the two groups $(\bar{X}+s,$ points).

Group	Before the intervention	On the day of transfer	On the third day following the transfer	Intergroup effects	Time effect	Interaction effect
				*F*	*F*	*F*
Experimental group	29.91 ± 6.30	23.23 ± 3.26	17.91 ± 2.18	89.523	13.273	89.738
Control group	29.80 ± 6.16	32.20 ± 4.78	34.77 ± 5.30			
*t*	0.086	−10.287	−19.511			
*P*	0.898	0.001	<0.001			

### 3.4 Comparison of CCFSS scores before and after intervention in the families of the two groups of children

Before the intervention, there was no statistically significant difference in the CCFSS scores between the families of the two groups of children (*p* > 0.05). After the intervention was implemented through hospital-based follow-up, on the day of transfer, the CCFSS score of the families of the children in the experimental group (66.73 ± 11.45) was higher than that of the control group (43.66 ± 15.58), and the difference was statistically significant (*p* < 0.05). On the third day after the transfer, the CCFSS score of the families of the children in the experimental group (80.91 ± 9.64) was higher than that of the control group (37.89 ± 14.49), and the difference was statistically significant (*p* < 0.05) (see Table 4).

**Table 4:** Comparison of CCFSS scores before and after the intervention among the families of the two groups $(\bar{X}+s,$ points).

Group	Before the intervention	On the day of transfer	On the third day following the transfer	Intergroup effects	Time effect	Interaction effect
				*F*	*F*	*F*
Experimental group	47.45 ± 16.59	66.73 ± 11.45	80.91 ± 9.64	60.302	55.128	183.243
Experimental group	47.27 ± 16.54	43.66 ± 15.58	37.89 ± 14.49			
*t*	0.051	7.916	16.398			
*P*	0.972	0.008	<0.001			

## 4 Discussion

### 4.1 In-hospital follow-up can effectively improve family post-ICU syndrome

PICS (Post-Intensive Care Syndrome) refers to a syndrome in which children and their families experience or continue to experience deterioration in emotional, cognitive, and psychosocial aspects, starting from admission to the PICU and lasting through to discharge and returning home (9, 10). PICS symptoms can appear as early as 24 h after ICU admission and may persist for 5 to 15 years after discharge (1, 9). Research indicates that PICU-S can impact the physical and mental health of the families of critically ill children, while also leading to a reduction in familial support for the child, which in turn can affect the child’s prognosis (11). Table 2 shows that the ICU syndrome scores of the family members of children in the in-hospital follow-up group On the day of Transfer to another department and 3 days after Transfer to another department were significantly lower than those in the control group. This is consistent with the findings of studies by Watland S et al. (12), Johanna Josepha Op’t Hoog SA et al. (13), and Putowski Z et al. (14), possible reason is the effective communication by medical staff, the establishment of a compassionate visitation model, and the full respect for and fulfillment of the family’s individual needs. This ensured that the family received relevant information, emotional support, the ability to communicate with medical staff at any time, and assurance that they were receiving the best medical care. These factors contributed to a reduction in PICU-S, facilitated the acquisition of disease-related knowledge and skills by the family, and improved their self-management capabilities regarding the illness. Table 2 shows that the repeated measures analysis of variance for the ICU syndrome scores of the family members of the two groups revealed significant differences in the group effect, time effect, and interaction effect, all of which were statistically significant (*p* < 0.05). Furthermore, the scores in the experimental group showed a continuous increase over the two time points, indicating that as the intervention time increased, the improvement in the ICU syndrome of the family members of the children became more pronounced. Therefore, in-hospital follow-up can effectively improve the PICU-S of the family members of the children.

### 4.2 In-hospital follow-up can reduce the depression-anxiety-stress emotions of the family members of the children

Anxiety, depression, and stress are the main manifestations of PICU-F psychological disorders, with anxiety and depressive symptoms being the most common (15). Studies abroad have shown that the incidence of anxiety among ICU pediatric patients’ family members ranges from 21 to 56%, and the incidence of depression ranges from 8 to 42% (16). Even after the child is transferred out of the ICU, symptoms such as anxiety and depression in the family members may persist. Table 3 shows that on the day of in-hospital follow-up and 3 days after Transfer to another department, the depression-anxiety-stress scores of the family members of the children in the experimental group were lower than those in the control group, which is consistent with the findings of Gunnlaugsdottir T et al. (17) and Davidson JE et al. (18). Possible reason is that as the child’s condition improves, the family members’ depression-anxiety-stress emotions may ease. However, after the transfer from a non-visitation room to a visitation room, the family members may still be concerned about the continuity of treatment and care for the child. Follow-up personnel indicated that they would continue to visit the visitation room for joint assessments with the family members 3 days after Transfer to another department, participating together in the treatment and care plan to ensure the continuity of the child’s treatment and care, thereby alleviating the family’s depression-anxiety-stress emotions. Table 3 shows that the repeated measures analysis of variance for the depression-anxiety-stress scores of the family members of both groups revealed significant differences in the between-group effect, time effect, and interaction effect, all of which were statistically significant (*p* < 0.05). Furthermore, the experimental group showed a continuous increase over the two time points, indicating that as the intervention time increased, the improvement in the family members’ depression-anxiety-stress emotions became more pronounced. Therefore, based on the fact that in-hospital follow-up is beneficial for improving the depression-anxiety-stress conditions of the family members of the children, healthcare professionals need to pay more attention to the psychological health of the patients’ families. They should conduct more monitoring, follow-up, and further psychological interventions for both the children and their families, thereby improving the psychological symptoms such as anxiety and depression in both the patients and their families, and continuously enhancing their quality of life.

### 4.3 In-hospital follow-up can improve the satisfaction of the children’s family members

Improving the satisfaction of the family members of children in the intensive care unit is an important nursing goal. The satisfaction of the patients’ families not only reflects the quality and effectiveness of medical services but also affects the recovery and quality of life of the children. Table 4 shows, that the satisfaction scores of the family members of children in the experimental group on the day of transfer and 3 days after Transfer to another department were higher than those in the control group. This is consistent with the findings of studies by Nassar Junior AP (19) and Zante B, et al. (20), the possible reason is the adherence to a people-centered service philosophy. By engaging in effective communication with the family members, negative emotions are regulated, psychological stress is alleviated, and the goal of enhancing the psychological resilience of the family members is achieved. This approach encourages the active involvement of the family, leading to better cooperation with treatment and nursing tasks, thereby improving the satisfaction of the family members with the nursing care. Table 4 shows that the repeated measures ANOVA of the satisfaction scores of the family members in both groups revealed significant differences in the between-group effect, time effect, and interaction effect, all of which were statistically significant (*p* < 0.05). Moreover, the satisfaction scores in the experimental group showed a continuous increase across the two time points, indicating that as the intervention period progressed, the satisfaction of the family members improved progressively. Therefore, based on in-hospital follow-up, it can effectively improve the satisfaction of the family members of children and help enhance the children’s quality of life.

## 5 Study limitations

However, this study only selected the family members of children from a single tertiary Grade A hospital as the research subjects, which may introduce some selection bias. The sample size is relatively small, and the follow-up period is short, so the results may be subject to some degree of bias. Future studies are recommended to expand the sample size, conduct multi-center research, and further validate the effectiveness of the intervention in the family members of children in the PICU.

## 6 Conclusion

In conclusion, in-hospital follow-up can effectively alleviate post-intensive care unit syndrome in the families of PICU patients, reduce negative emotions such as depression, anxiety, and stress, improve family satisfaction.

## Data Availability

The raw data supporting the conclusions of this article will be made available by the authors, without undue reservation.
